# Identification of metastasis and prognosis-associated genes for serous ovarian cancer

**DOI:** 10.1042/BSR20194324

**Published:** 2020-06-25

**Authors:** Yijun Yang, Suwan Qi, Can shi, Xiao Han, Juanpeng Yu, Lei Zhang, Shanshan Qin, Yingchun Gao

**Affiliations:** 1 Department of Obstetrics and Gynecology, The Affiliated Huai'an No. 1 People's Hospital of Nanjing Medical University, Huai'an, China; 2 Department of Central Laboratory, The Affiliated Huai'an No. 1 People's Hospital of Nanjing Medical University, Huai'an, China

**Keywords:** bioinformatics analysis, prognosis, serous ovarian cancer, TIMER database analysis, tumor metastasis, weighted gene coexpression network analysis (WGCNA)

## Abstract

Serous ovarian cancer is one of the most fatal gynecological tumors with an extremely low 5-year survival rate. Most patients are diagnosed at an advanced stage with wide metastasis. The dysregulation of genes serves an important role in the metastasis progression of ovarian cancer. Differentially expressed genes (DEGs) between primary tumors and metastases of serous ovarian cancer were screened out in the gene expression profile of GSE73168 from Gene Expression Omnibus (GEO). Cytoscape plugin cytoHubba and weighted gene co-expression network analysis (WGCNA) were utilized to select hub genes. Univariate and multivariate Cox regression analyses were used to screen out prognosis-associated genes. Furthermore, the Oncomine validation, prognostic analysis, methylation mechanism, gene set enrichment analysis (GSEA), TIMER database analysis and administration of candidate molecular drugs were conducted for hub genes. Nine hundred and fifty-seven DEGs were identified in the gene expression profile of GSE73168. After using Cytoscape plugin cytoHubba, 83 genes were verified. In co-expression network, the blue module was most closely related to tumor metastasis. Furthermore, the genes in Cytoscape were analyzed, showing that the blue module and screened 17 genes were closely associated with tumor metastasis. Univariate and multivariate Cox regression revealed that the age, stage and *STMN2* were independent prognostic factors. The Cancer Genome Atlas (TCGA) suggested that the up-regulated expression of *STMN2* was related to poor prognosis of ovarian cancer. Thus, *STMN2* was considered as a new key gene after expression validation, survival analysis and TIMER database validation. GSEA confirmed that *STMN2* was probably involved in ECM receptor interaction, focal adhesion, TGF beta signaling pathway and MAPK signaling pathway. Furthermore, three candidate small molecule drugs for tumor metastasis (diprophylline, valinomycin and anisomycin) were screened out. The quantitative reverse transcription-polymerase chain reaction (qRT-PCR) and western blot showed that *STMN2* was highly expressed in ovarian cancer tissue and ovarian cancer cell lines. Further studies are needed to investigate these prognosis-associated genes for new therapy target.

## Introduction

As one of the most prevalent malignancies in gynecology, ovarian cancer carries the highest mortality [1]. Most patients are diagnosed at an advanced stage with wide metastasis, leading to an extremely low 5-year survival rate. Tumor metastasis is closely related with poor prognosis, and is also the main death reason in patients with ovarian cancer [2,3]. Therefore, it is vital to explore new biomarkers to suppress tumor metastasis.

Because of the limitation of molecular biology research, bioinformatics has been widely utilized in screening and analyzing the genes related to occurrence and progression of tumor [4,5]. A lot of genes with similar expression pattern actually function within a whole network and affect each other [6]. Most of previous studies have only identified the single gene or protein, but do not describe the association between genes and interaction pathways. Weighted gene co-expression network analysis (WGCNA) is a biological network to describe the correlations between differentially expressed genes (DEGs) [7]. This method identifies synergistically altered genomes and specific candidate biomarkers based on the correlations between the genes and phenotype. WGCNA has been widely used to screen genes associated with the phenotype of cancer, such as cervical cancer and pancreatic ductal adenocarcinoma [8,9]. In the present paper, the DEGs were analyzed and biological network was constructed to verify hub genes implied in the metastasis of ovarian cancer.

## Methods and materials

### Microarray data

To explore significant genes in linkage to the metastasis progression of ovarian carcinoma, the raw gene expression profiles of GSE73168 dataset were acquired from Gene Expression Omnibus (GEO) database (https://www.ncbi.nlm.nih.gov/geo/). The mRNA expression profile of GSE73168 dataset was detected by using GPL570 platform (Affymetrix Human Genome U133 Plus 2.0 Array). Twenty-four samples from eight patients were involved in the GSE73168 profile, including primary tumor tissue samples with matching ascites tumor cell samples and metastatic tumor samples.

### Identification of differentially expressed genes

The flow diagram for the design of this research is displayed in Supplementary Figure S1. The raw dataset of GS73168 was analyzed with the GEO2R (https://www.ncbi.nlm.nih.gov/geo/geo2r/), a web tool using ‘limma’ package of R and utilized to screen DEGs between primary and metastasis tumor tissues. The points with |logFC| ≥ 1 and *P*-value < 0.05 were defined to be statistically meaningful cut-off points. Gene annotation and corresponding data files of the DEGs were extracted through R software.

### Gene Ontology analysis and Kyoto Encyclopedia of Genes and Genomes analysis

To further systematically analyze the functional annotation of DEGs, the clusterProfiler package of R [10] was utilized to perform [11] Gene Ontology (GO) and Kyoto Encyclopedia of Genes and Genomes (KEGG) analysis. The *P*-value was conventionally set at 0.05.

### Protein–protein interaction network construction

To explore interactions between DEGs, a protein–protein interaction (PPI) was constructed by using the Search Tool of the Retrieval of Interacting Genes Database (STRING) (https://www.string-db.org/), an authoritative database to assess interactions between proteins. The cut-off value was set at a confidence score >0.7 and individual nodes were filtered out.

### Screening of hub module

String interactions were imported into Cytoscape plug-in cytoHubba to select the hub genes of biological network analysis [12]. In our study, the top 20 genes were chosen as hub genes.

### Co-expression and module functional analysis

Co-expression analysis is a multidirectional network, in which each node represents a gene. The DEGs of GSE73168 dataset were utilized to construct co-expression network after assessing the expression profile. The R package ‘WGCNA’ was used to build co-expression network of DEGs. In addition, Pearson's correlation coefficient of genes was calculated to obtain similarity matrix. To make the network conform to scale-free network distribution, an appropriate weight value was selected and calculated. An appropriate soft threshold value was chosen to measure the connectivity between genes. The adjacency matrix was converted into topological overlap matrix (TOM); meanwhile, the WGCNA package was used to perform hierarchical clustering on the matrix. For the generated clustering tree, Dynamic Tree Cut method was adopted to cut the gene clustering tree. Genes with similar expression patterns were allocated to a branch, and each branch represented a co-expression module. In present study, the soft threshold was 7 and the minimum size of module was set as 30. After analyzing the association between modules and clinical traits, the hub module was selected, which is the most relevant to metastasis in ovarian cancer. Gene significance (GS) represents the association between the gene and clinical traits. Module membership (MM) reveals the relation between module eigen genes and gene expression.

### Venn diagram of hub genes

The intersection of PPI hub genes and blue module genes were considered as real hub genes, which are probably associated with metastasis. To achieve that intersection, an online web tool (http://bioinfogp.cnb.csic.es/tools/venny/index.html) was used to plot Venn diagram.

### Prognostic signature of hub gene

The RNA sequencing data and the matching clinical characteristic data of patients with serous ovarian cancer were obtained from The Cancer Genome Atlas (TCGA) database (https://Cancergenome.nih.gov/). R package ‘survival’ was utilized to conduct overall survival analysis. The Univariate Cox regression analysis was performed to investigate the correlation between gene expression and overall survival. Genes with *P*-value <0.05 were considered to be significant.

### Validations and analysis of hub gene

Hub genes were further analyzed by using the data downloaded from Illumina Human Methylation 27 platform in the TCGA database. The ONCOMINE database [13] (www.Oncomine.org), a publicly accessible online cancer database integrating sequencing data from several database, was used to assess different expressions of *STMN2* in ovarian cancer tissues and ovarian normal tissues.

### Gene set enrichment analysis

The samples of GSE73168 dataset have been placed into two groups according to the expression levels of hub genes. Gene set enrichment analysis (GSEA) (https://software.broadinstitute.org/gsea/index.jsp) was conducted in order to explore biological function. Terms with *P* < 0.05 were identified to be significant.

### TIMER database analysis

Tumor Immune Estimation Resource (TIMER) is an integrated investigation for molecular characterization of immune infiltration (https://cistrome.shinyapps.io/timer/) [14]. TIMER uses six previously published statistical modules to study the correlation between the gene expression and immune cell tumor-infiltration [15]. Gene expression levels were visualized with log 2 RSEM.

### Analysis of small molecule drugs

The Connectivity Map (CMap) database was established to explore the small molecule involved in tumor metastasis [16]. The DEGs were inputted into CMap database. The enrichment scores ranging from −1 to 1 were analyzed. The negative linkage score indicates that the drugs can reverse input characteristics. The connectivity score was evaluated by the number of instances (*N* > 10) and *P-*value <0.05. The tomography of small molecule drugs was displayed by using the Pubchem database (https://pubchem.ncbi.nlm.nih.gov/).

### Patients and tissue samples

Twenty fresh ovarian tissue samples (10 normal and 10 ovarian cancers) were frozen in the liquid nitrogen after washed by ice-cold PBS once removed from patients. In the present study, the usage of patients’ tissues and access to patients’ information were approved by Ethics Committee of the First Affiliated Huai'an Hospital of Nanjing Medical University.

### Cell culture

The human normal ovarian epithelial immortalized cell line IOSE-80 was cultivated in M199 medium (KeyGEN, Nanjing, China) supplemented with 10% fetal bovine serum (FBS, AusGeneX, AUS). The human ovarian cancer lines SK-OV3, HO-8910 and A2780 (Shanghai Institute of Cell Biology) were cultivated in the McCoy's 5A medium (KeyGEN, Nanjing, China) and RPMI-1640 medium (Gibco BRL, Grand Island, U.S.A.) supplemented with 10% FBS in incubator containing 5% CO_2_ at 37°C.

### RNA extraction and quantitative polymerase chain reaction

RNA isolation of fresh ovarian tissue samples were conducted through TRIzol Reagent (Ambion, U.S.A.). The synthesis of complementary DNA (cDNA) was conducted using HiScript III RT SuperMix kit (Vazyme, Nanjing, China) through reverse transcription reaction. Quantitative polymerase chain reaction for *STMN2* and *β-actin* were conducted using ChamQ SYBR qPCR Master Mix kit (Vazyme, Nanjing, China). *β‐actin* was selected as the internal reference gene. The primer sequences were as follows: *STMN2* forward 5′-GCTCTTGCTTTTACCCGGAAC-3′; *STMN2* reverse 5′-AGGCACGTTTGTTGATTTGCT-3′; *β-actin* forward 5′-CATGTACGTTGCTATCCAGGC-3′; *β-actin* reverse 5′-CTCCTTAATGTCACGCACGAT-3′.

### Western blot experiment

RIPA buffer (Tris 50 mmol/l, NaCl 150 mmol/l, EDTA 0.5 mmol/l, NP40 1%, Triton X-100 0.5%, glycerin 10%) supplemented with protease inhibitor cocktail (MCE, Shanghai, China) and phosphatase inhibitors was used to extract proteins from cells and fresh ovarian tissues. After using Bicinchoninic Acid Kit (Beyotime, Shanghai, China) for protein quantification, the lysate was metallized with added loading buffer at 100°C for 10 min to denature. The protein was transferred to the PVDF membrane after sodium dodecyl sulfate/polyacrylamide gel electrophoresis. After blocked with 5% defatted milk powder dissolved in TBST (pH 7.4, NaCl 8 g/l, KCl 0.2 g/l, Tris 3 g/l) at room temperature for 1 h, the membrane was incubated with the primary antibody at 4°C overnight, including *STMN2* (1:1000, ab185956, Abcam, U.S.A.) and *β-actin* (1:1000, A5441, Sigma, U.S.A.). The membrane was incubated with horseradish peroxidase-coupled secondary antibody at room temperature for 1 h after washed with TBST for four times. Target protein bands were developed by Super ECL Plus kit (US Everbright INC, U.S.A.) and quantitatively analyzed by Image J.

### Statistical analysis

Statistical analysis was conducted using Graphpad Prism 6. Unpaired *t* test was utilized for comparing continuous variables between two groups. *P*-value less than 0.05 was considered to be statistically significant.

## Results

### Identification and enrichment of DEGs in ovarian cancer

**Figure 1:**
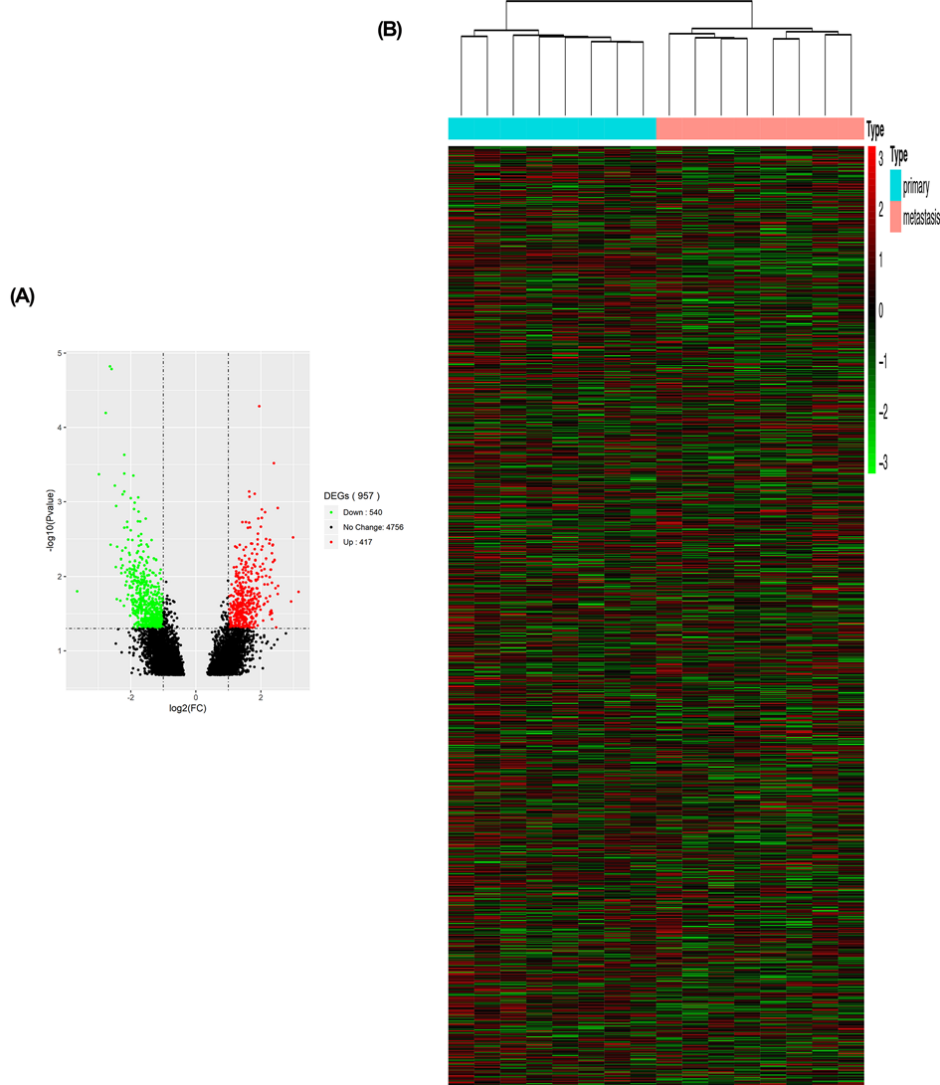
DEGs identified in GSE73168 (**A**) Volcano map of DEGs between primary tumors and metastases of serous ovarian cancer. The red plots in the volcano represent up-regulation and the green points represent down-regulation. (**B**) Heatmap of the all DEGs according to the value of |log FC|. The color in heat maps from green to red shows the progression from low expression to high expression cell. log FC: log fold change.

The GEO2R tool was utilized to analyze DEGs in GSE73168. *P* <0.05 and |logFC| ≥1 were included as the standards. 957 DEGs between primary sites and metastases of serous ovarian cancer were filtered, revealing 417 up-regulated genes and 540 down-regulated genes. DEGs were displayed in the volcano map and heatmap based on the |logFC| values (Figure 1). DEGs were mostly enriched in cell fate commitment, cell fate specification, neuron fate specification, neuron fate commitment, anion transmembrane transporter activity, chloride transmembrane transporter activity, chloride channel activity, inorganic anion transmembrane transporter activity, small GTPase binding, and anion channel activity in the GO analysis (Suppementary Figure S2A). In the KEGG analysis, DEGs were enriched in neuroactive ligand–receptor interaction, GABAergic synapse, and nicotine addiction (Suppementary Figure S2B).

### Hub module selection

**Figure 2:**
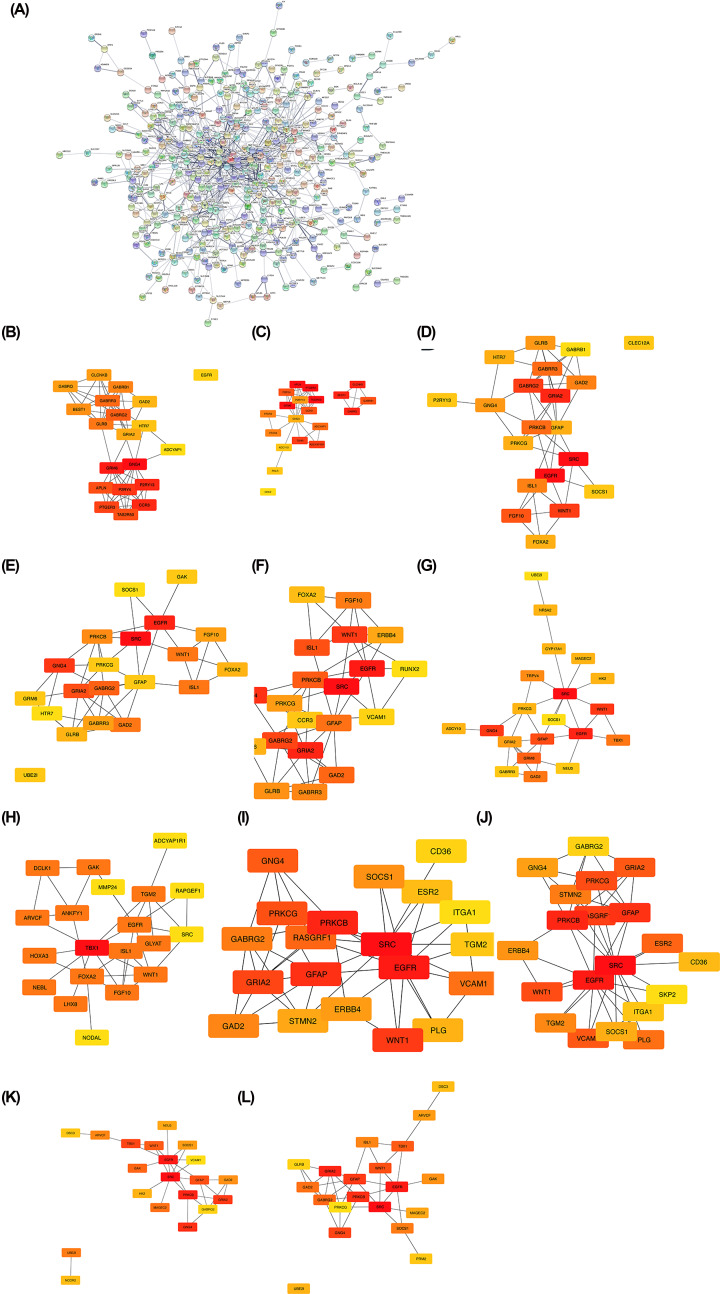
CytoHubba analysis of PPI network (**A**) 957 DEGs were filtered into the DEGs PPI network complex that contained 514 node and 842 side. (**B**) Maximal Clique Centrality methods in cytoHubba. (**C**) Betweenness methods in cytoHubba. (**D**) Bottleneck methods in cytoHubba. (**E**) Closeness methods in cytoHubba. (**F**) Degree methods in cytoHubba. (**G**) Density of maximum neighborhood component methods in cytoHubba. (**H**) EcCentricity methods in cytoHubba. (**I**) Edge percolated component methods in cytoHubba. (**J**) Maximum neighborhood component methods in cytoHubba. (**K**) Radiality methods in cytoHubba. (**L**) Stress methods in cytoHubba.

Through the STRING analysis, 957 DEGs were inputted into the PPI network, including 514 nodes and 842 sides (Figure 2A). Then cytoHubba, which is a plugin to rank nodes by their network capabilities [12], was utilized to offer 11 analysis methods including Density of Maximum Neighborhood Component, Maximal Clique Centrality, Maximum Neighborhood Component, Degree, Edge Percolated Component, and six centralities (Bottleneck, EcCentricity, Closeness, Radiality, Betweenness, and Stress). All of 11 analysis methods in the PPI network were used in the present study to identify top twenty genes (Figure 2B–L). Eighty-two genes were selected according to 11 analysis methods in CytoHubba. This finding may indicate that 82 genes play an important role in ovarian cancer.

### Weighted gene coexpression network construction and analysis

**Figure 3:**
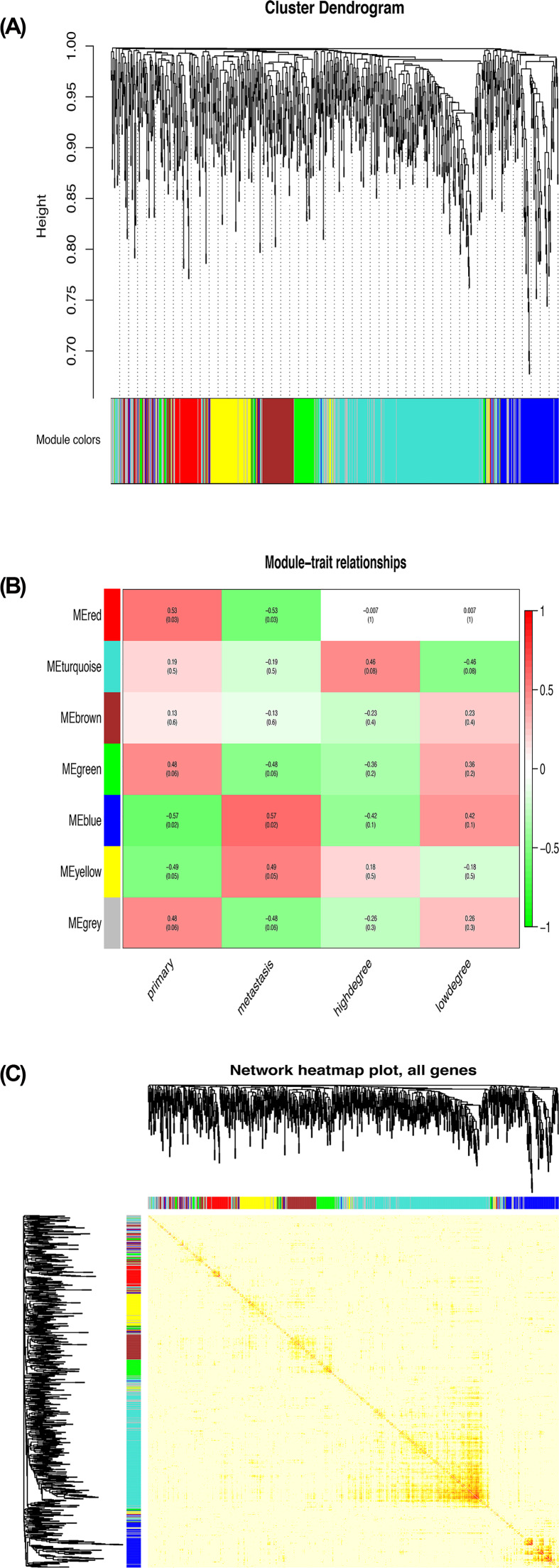
Hub module selection (**A**) Dendrogram of all DEGs clustered based on a dissimilarity measure (1-TOM). (**B**) Correlation between modules and traits. The upper number in each cell refers to the correlation coefficient of each module in the trait, and the lower number is the corresponding *P*-value. Among them, the blue module was the most relevant modules with cancer traits. (**C**) A heatmap of all genes. The intensity of the red color indicates the strength of the correlation between pairs of modules on a linear scale.

A total of 16 clinical samples of GSE73168 were collected for analysis (Supplementary Figure S1B). The ‘WGCNA’ package in R was conducted, and the genes with highly related genes were grouped into one module. β = 7 (scale free *R*^2^ = 0.90) was chosen to be appropriate soft-thresholding value in order to ensure a scale-free analysis (Supplementary Figure S3). Seven modules were identified, and it was found that blue module was mostly related to tumor metastasis (Figure 3A,B). All the genes were covered in the heat map (Figure 3C). Furthermore, intra-module analysis of GS and MM in seven modules was performed. GS and MM were significantly associated, implying that 112 genes involved in blue module probably have significant association with metastasis among seven modules (Supplementary Figure S4A).

Interestingly, similarities were observed in certain gene modules. To find out the connectivity among the seven gene modules, interactions of eigengenes were further analyzed. Seven clusters were again divided into two clusters, including two branches (Supplementary Figure S4B,C).

### Hub genes identification

**Figure 4:**
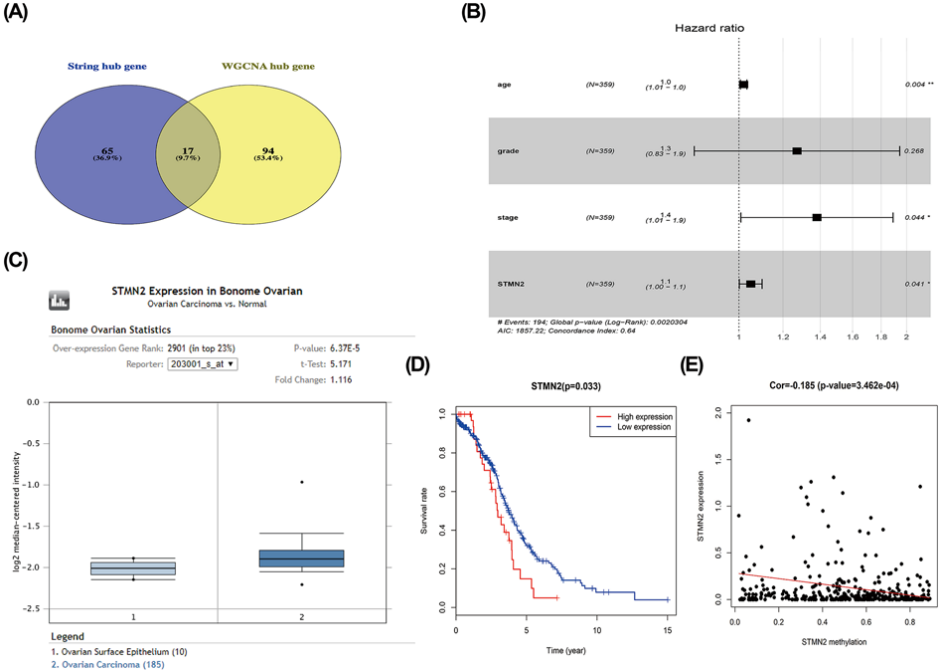
Hub genes analysis (**A**) Real key genes belonging to both the blue module and the PPI network. (**B**) Univariate Cox regression analysis to predict prognostic factors associated with patient survival. (**C**) Expression boxplots of gene STMN2 in Oncomine database. (**D**) Survival analysis of STMN2. (**E**) The expression of *STMN2* was negatively correlated with DNA methylation.

One hundred twelve genes in the blue module were chosen as hub genes. Eighty-two genes identified by cytoHubba in the PPI network were defined as hub genes. Finally, intersection of PPI network and WGCNA analysis were 17 genes, which were considered to be ‘real’ hub genes (Figure 4A).

### Prognostic analysis of hub gene

Next, the prognostic signature of 17 hub genes were analyzed. Univariate and multivariate Cox regression analyses were employed to predict factors related with the prognosis (Table 1). The age, stage, and *STMN2* expression were significantly correlated to overall patient survival using TCGA database, except grade (Figure 4B). Univariate and multivariate Cox regression analyses of the remaining 16 genes implied no correlation with overall patient survival (Supplementary Table S1).

**Table 1:** Univariate and multivariate Cox regression analysis for STMN2 and clinical features

	Univariate analysis			Multivariate analysis		
Variables	HR	95% CI	*P*-value	HR	95% CI	*P*-value
Age	1.020	1.007–1.034	0.0036*	1.020	1.006–1.033	0.0041*
Grade	1.378	0.904–2.102	0.1360	1.271	0.832–1.944	0.2676
Stage	1.391	1.023–1.892	0.0351*	1.381	1.008–1.891	0.0443*
STMN2	1.345	1.027–1.762	0.0312*	1.050	1.002–1.101	0.0412*

HR, hazard ratio.

* *P*<0.05

### Hub gene validation

*STMN2* expression was further verified in ovarian cancer by using FireBrowse and ONCOMINE database. ONCOMINE analysis showed that *STMN2* transcripts were 1.116-fold higher in cancer compared with normal samples from Bonome Ovarian Statistics (*P* = 6.37E−5), indicating that the expression of *STMN2* was up-regulated in ovarian cancer (Figure 4C). The gene was further studied to understand the possible mechanism for abnormal expression. By using the Illumina Human Methylation 27 platform, the expression of *STMN2* was negatively correlated with DNA methylation (Figure 4E). Survival analysis implied that higher *STMN2* expression was correlated with poorer survival rate (Figure 4D).

### Gene set enrichment analysis

To explore underlying mechanism of *STMN2* in ovarian cancer, GSEA analysis was performed to identify the enriched KEGG pathways. Four gene sets (*n* = 75), ‘ECM receptor interaction’, ‘focal adhesion’, ‘TGF beta signaling pathway’, and ‘MAPK signaling pathway’ were enriched (*P* < 0.05) (Supplementary Figure S6A–D).

### Pertinence of *STMN2* expression and immune infiltration level in ovarian cancer

**Figure 5:**
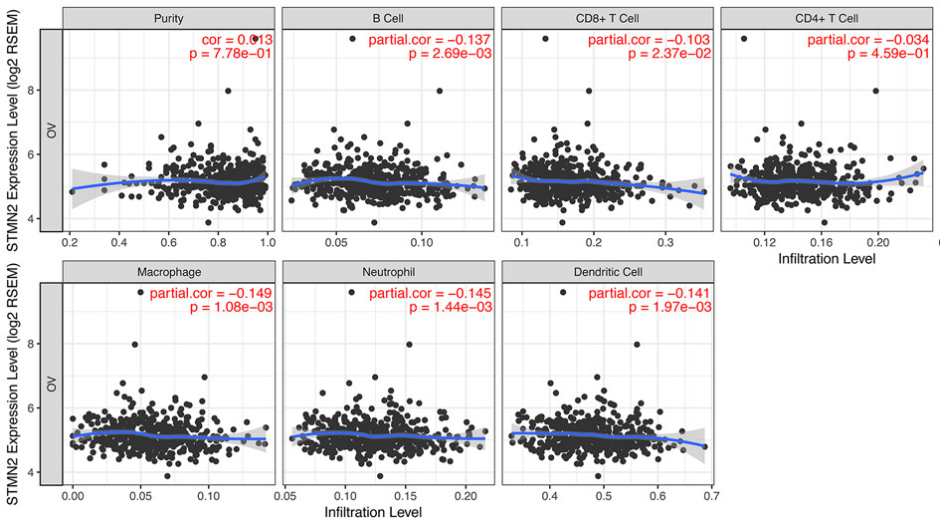
STMN2 expression negatively correlates with immune cell infiltration levels in ovarian cancer through TIMER Correlation between STMN2 expression and the abundances of six immune infiltrates (B cells, CD4+ T cells, CD8+ T cells, neutrophils, macrophages, and dendritic cells) are displayed. The purity-corrected partial Spearman correlation and statistical significance are shown on the top right corners.

The distribution of tumor-infiltrating lymphocytes is an important indicator of patients’ lymph node status and prognosis[17,18]. The association of *STMN2* expression level with immune infiltration abundance in ovarian tumor was evaluated using TIMER database. *STMN2* expression was negatively correlated with infiltration degree of B cells, CD8+ T cells, macrophage, neutrophil, and dendritic cells (Figure 5).

### Related small molecule drug screening

**Figure 6:**
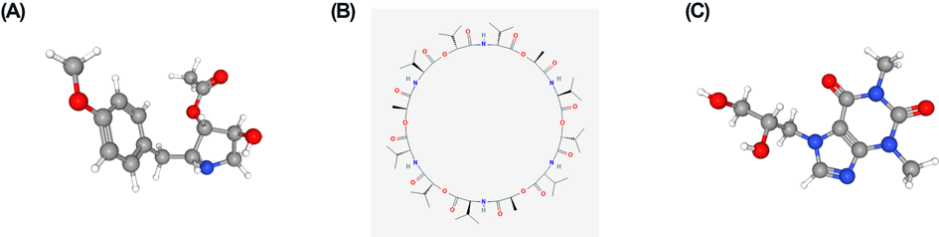
The tomographs of the three candidate small molecule drugs for the metastasis (**A**) Diprophylline, (**B**) valinomycin, and (**C**) anisomycin.

All DEGs were explored in CMap database to analyze the small molecule drugs. Three small molecule drugs with high connectivity scores are displayed in Table 2. Diprophylline, valinomycin, and anisomycin showed a negative association with the tumor metastasis and implied great possibility in clinical application. The tomography of the three molecules was displayed in the PubChem database (Figure 6).

**Table 2:** The top three metastasis-related small molecules with highly significant correlations in results of CMap analysis

CMap name	Mean	*N*	Enrichment	*P* value
Diprophylline	−0.511	5	−0.703	0.00505
Valinomycin	−0.339	4	−0.641	0.03991
Anisomycin	−0.41	4	−0.631	0.0455

### Positive expression of *STMN2* in ovarian cancer

**Figure 7:**
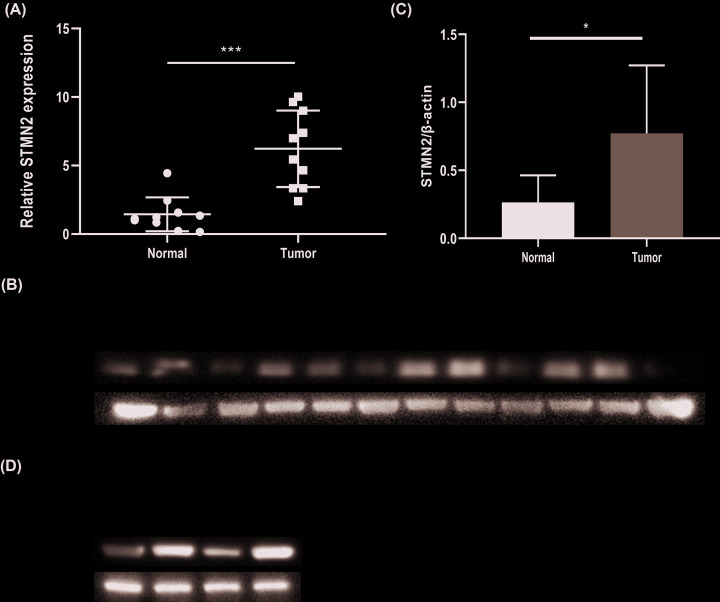
STMN2 is high expression in ovarian cancer tissue and cell lines (**A**) qRT-PCR showed that STMN2 was overexpressed in ovarian cancer tissue campared with normal ovarian tissue. (**B,C**) Western blot experiment showed that STMN2 was overexpressed in ovarian cancer tissue. (**D**) Western blot showed high expression of STMN2 in ovarian cancer cell lines.

The high expression of *STMN2* in ovarian cancer tissue was verified in mRNA and protein level compared with normal ovarian tissue, which is consistent with the results of Oncomine database (Figure 7A–C). Furthermore, the expression level of *STMN2* in ovarian cell lines was also explored through western blot. *STMN2* was highly expressed in ovarian cancer cell lines (SK-OV3, A2780, and HO-8910) compared with normal cell lines (IOSE-80) (Figure 7D).

## Discussion

Ovarian cancer is the prevalent malignancy of female reproductive tract. The high rate of recurrence and metastasis leads to an extremely low 5-year survival rate. Hence, it is vital to screen the markers for early diagnosis and regulatory pathways involved in metastasis of ovarian cancer. Gene expression profile of GSE73168 containing eight primary tumors and eight matched metastases of serous ovarian cancer was utilized to seek some biomarkers and figure out the molecule mechanism of metastasis in ovarian cancer in this research.

957 DEGs were found to be related to tumor metastasis, including 417 up-regulated genes and 540 down-regulated genes. The PPI and WGCNA analyses were used to choose the hub genes related to clinical metastasis. In addition, the analysis of functions and pathways involved in ovarian cancer was performed.

In the GO analysis, DEGs were mostly enriched in the cell fate commitment, cell fate specification, neuron fate specification, neuron fate commitment, anion transmembrane transporter activity, chloride transmembrane transporter activity, chloride channel activity, inorganic anion transmembrane transporter activity, small GTPase binding, and anion channel activities related to the cell cycle, which is consistent with characteristics of rapid proliferation and fast growing in ovarian cancer [19]. In the KEGG, DEGs were mostly enriched in neuroactive ligand–receptor interaction, GABAergic synapse, and nicotine addiction. Neuroactive ligand–receptor interaction has been demonstrated to be involved in some cancers, such as renal cell carcinoma [20], breast cancer [21], and lung adenocarcinoma [22].

WGCNA analysis indicated that seven modules possessed related expression patterns. 112 hub genes in the blue module connected with metastasis were selected. Based on the plugin cytoHubba in Cytoscape, 17 genes were screened. Univariate Cox regression of 17 genes was analyzed by the TCGA database, and *STMN2* was finally obtained.

The expression of *STMN2* was up-regulated in cancer group compared with the normal group. *STMN2* was highly expressed in ovarian cancer tissue and cell lines. *STMN2* is a member of the stathmin gene family, and plays an important part in human hepatoma cells [23,24]. Stathmin gene family members participate in microtubule polymerization [25]. And it was reported that *STMN2* is a new target of β-catenin/TCF-mediated transcription in hepatoma cell [23]. *STMN2* is involved in TCF binding sites and participates in Wnt/β-catenin signaling [24].

Furthermore, our research showed that *STMN2* expression levels were associated with CD4+ T cell, B cell, CD8+ T cell, macrophage, neutrophil and dendritic cell. Immune-related mechanisms involved in some cancer and immunotherapy strategy is a potential direction for the treatment of ovarian cancer [26]. This result indicated that *STMN2* participates in the recruitment and regulation of immune-infiltrating cells in ovarian cancer. However, functions and pathways of *STMN2* in tumor-infiltrating lymphocytes need further study.

Nowadays, paclitaxel/carboplatin combined with chemotherapy has been widely used for ovarian cancer patients. In this research, three small molecule drugs were identified, which may serve as potential therapeutic targets. Diprophylline, the most important small molecule in our study, has been reported to suppress proliferation and migration of non-small cell lung cancer via down-regulating PI3K signaling pathway [27]. Valinomycin shows a potential therapeutic effect when combined with cisplatin *in vitro* [28]. Anisomycin has been revealed to suppress proliferation and invasion of ovarian cancer stem cell through regulating LncRNA BACE1-AS [29].

## Conclusion

In conclusion, through an integrated bioinformatics analysis, *STMN2* was screened as a hub gene, mostly associated with the metastasis of ovarian cancer, and its functions and pathways involved in the ovarian cancer were explored. The gene deserves further exploration and analysis in combination with the clinical conditions of the patients. Further studies to explore functions and mechanism of *STMN2* in ovarian cancer metastatic process will be performed in our future work.

## Supplementary Material

Supplementary Figures S1-S6 and Table S1

## Data Availability

The data used in the current study are available from the Gene Expression Omnibus (https://www.ncbi.nlm.nih.gov) and Cancer Genome Atlas database (https://cancergenome.nih.gov).

## Abbreviations

CMap: connectivity map
DEG: differentially expressed gene
GEO: Gene Expression Omnibus
GO: Gene Ontology
GS: gene significance
GSEA: gene set enrichment analysis
KEGG: Kyoto Encyclopedia of Genes and Genomes
logFC: log fold change
MM: Module Membership
PPI: protein–protein interaction
TCGA: The Cancer Genome Atlas
TOM: topological overlap matrix
WGCNA: weighted gene co-expression network analysis

## References

[1] Siegel R.L., Miller K.D. and Jemal A. (2019) Cancer statistics, 2019. CA Cancer J. Clin. 69, 7–34 10.3322/caac.21551 30620402

[2] Jimenez-Sanchez A., Memon D., Pourpe S. et al. (2017) Heterogeneous tumor-immune microenvironments among differentially growing metastases in an ovarian cancer patient. Cell 170, 927.e920–938.e920 10.1016/j.cell.2017.07.025 28841418 PMC5589211

[3] Matz M., Coleman M.P., Carreira H. et al. (2017) Worldwide comparison of ovarian cancer survival: histological group and stage at diagnosis (CONCORD-2). Gynecol. Oncol. 144, 396–404 10.1016/j.ygyno.2016.11.019 27919574 PMC6195190

[4] Xia L., Su X., Shen J. et al. (2018) ANLN functions as a key candidate gene in cervical cancer as determined by integrated bioinformatic analysis. Cancer Manag. Res. 10, 663–670 10.2147/CMAR.S162813 29670400 PMC5896649

[5] Yuan L., Zeng G., Chen L. et al. (2018) Identification of key genes and pathways in human clear cell renal cell carcinoma (ccRCC) by co-expression analysis. Int. J. Biol. Sci. 14, 266–279 10.7150/ijbs.23574 29559845 PMC5859473

[6] Yin L., Cai Z., Zhu B. et al. (2018) Identification of key pathways and genes in the dynamic progression of HCC based on WGCNA. Genes (Basel) 9, 10.3390/genes9020092 PMC5852588 29443924

[7] Zhang J., Lan Q. and Lin J. (2018) Identification of key gene modules for human osteosarcoma by co-expression analysis. World J. Surg. Oncol. 16, 89 10.1186/s12957-018-1381-y 29720180 PMC5932805

[8] Zhou Z., Cheng Y., Jiang Y. et al. (2018) Ten hub genes associated with progression and prognosis of pancreatic carcinoma identified by co-expression analysis. Int. J. Biol. Sci. 14, 124–136 10.7150/ijbs.22619 29483831 PMC5821034

[9] Yao S. and Liu T. (2018) Analysis of differential gene expression caused by cervical intraepithelial neoplasia based on GEO database. Oncol. Lett. 15, 8319–8324 29805564 10.3892/ol.2018.8403 PMC5950031

[10] Yu G., Wang L.G., Han Y. et al. (2012) clusterProfiler: an R package for comparing biological themes among gene clusters. OMICS 16, 284–287 10.1089/omi.2011.0118 22455463 PMC3339379

[11] Peng J., Wang H., Lu J. et al. (2017) Identifying term relations cross different gene ontology categories. BMC Bioinformatics 18, 573 10.1186/s12859-017-1959-3 29297309 PMC5751813

[12] Chin C.H., Chen S.H., Wu H.H. et al. (2014) cytoHubba: identifying hub objects and sub-networks from complex interactome. BMC Syst. Biol. 8, S11 10.1186/1752-0509-8-S4-S11 25521941 PMC4290687

[13] Rhodes D.R., Kalyana-Sundaram S., Mahavisno V. et al. (2007) Oncomine 3.0: genes, pathways, and networks in a collection of 18,000 cancer gene expression profiles. Neoplasia 9, 166–180 10.1593/neo.07112 17356713 PMC1813932

[14] Li T., Fan J., Wang B. et al. (2017) TIMER: a web server for comprehensive analysis of tumor-infiltrating immune cells. Cancer Res. 77, e108–e110 10.1158/0008-5472.CAN-17-0307 29092952 PMC6042652

[15] Li B., Severson E., Pignon J.C. et al. (2016) Comprehensive analyses of tumor immunity: implications for cancer immunotherapy. Genome Biol. 17, 174 10.1186/s13059-016-1028-7 27549193 PMC4993001

[16] Iorio F., Shrestha R.L., Levin N. et al. (2015) A semi-supervised approach for refining transcriptional signatures of drug response and repositioning predictions. PLoS One 10, e0139446 10.1371/journal.pone.0139446 26452147 PMC4599732

[17] Ko Y.S. and Pyo J.S. (2019) Clinicopathological significance and prognostic role of tumor-infiltrating lymphocytes in colorectal cancer. Int. J. Biol. Markers 34, 132–138 10.1177/1724600818817320 30852949

[18] Azimi F., Scolyer R.A., Rumcheva P. et al. (2012) Tumor-infiltrating lymphocyte grade is an independent predictor of sentinel lymph node status and survival in patients with cutaneous melanoma. J. Clin. Oncol. 30, 2678–2683 10.1200/JCO.2011.37.8539 22711850

[19] Dalton S. (2015) Linking the cell cycle to cell fate decisions. Trends Cell Biol. 25, 592–600 10.1016/j.tcb.2015.07.007 26410405 PMC4584407

[20] Liu X., Wang J. and Sun G. (2015) Identification of key genes and pathways in renal cell carcinoma through expression profiling data. Kidney Blood Press. Res. 40, 288–297 10.1159/000368504 26043775

[21] Huan J., Wang L., Xing L. et al. (2014) Insights into significant pathways and gene interaction networks underlying breast cancer cell line MCF-7 treated with 17beta-estradiol (E2). Gene 533, 346–355 10.1016/j.gene.2013.08.027 23978611

[22] Wu X., Zang W., Cui S. et al. (2012) Bioinformatics analysis of two microarray gene-expression data sets to select lung adenocarcinoma marker genes. Eur. Rev. Med. Pharmacol. Sci. 16, 1582–1587 23111975

[23] Lee H.S., Lee D.C., Park M.H. et al. (2006) STMN2 is a novel target of beta-catenin/TCF-mediated transcription in human hepatoma cells. Biochem. Biophys. Res. Commun. 345, 1059–1067 10.1016/j.bbrc.2006.05.017 16712787

[24] Lee H.S., Park M.H., Yang S.J. et al. (2007) Novel candidate targets of Wnt/beta-catenin signaling in hepatoma cells. Life Sci. 80, 690–698 10.1016/j.lfs.2006.10.024 17157329

[25] Peng H., Derrick B.E. and Martinez J.L. Jr. (2004) Time-course study of SCG10 mRNA levels associated with LTP induction and maintenance in the rat Schaffer-CA1 pathway in vivo. Brain Res. Mol. Brain Res. 120, 182–187 10.1016/j.molbrainres.2003.10.009 14741408

[26] Pan J.H., Zhou H., Cooper L. et al. (2019) LAYN is a prognostic biomarker and correlated with immune infiltrates in gastric and colon cancers. Front. Immunol. 10, 6 10.3389/fimmu.2019.00006 30761122 PMC6362421

[27] Zhao H.Y., Ren Y.H., Ren X.B. et al. (2019) Diprophylline inhibits non-small cell lung cancer A549 cell proliferation and migration, and promotes apoptosis, by downregulating PI3K signaling pathway. Oncol. Lett. 17, 857–862 30655839 10.3892/ol.2018.9678 PMC6312961

[28] Daoud S.S. and Sakata M.K. (1993) In vitro interaction of liposomal valinomycin and platinum analogs: cytotoxic and cytokinetic effects. Anticancer Drugs 4, 479–486 10.1097/00001813-199308000-00009 8400351

[29] Chen Q., Liu X., Xu L. et al. (2016) Long non-coding RNA BACE1-AS is a novel target for anisomycin-mediated suppression of ovarian cancer stem cell proliferation and invasion. Oncol. Rep. 35, 1916–1924 10.3892/or.2016.4571 26783004

