# Supplementary material for: Identification of metastasis and prognosis-associated genes for serous ovarian cancer
Source: Biosci Rep. 2020 Jun 25;40(6):BSR20194324. doi: 10.1042/BSR20194324 (PMC7317593; doi:10.1042/BSR20194324)
Supplement: Supplementary Figures S1-S6 and Table S1 [file BSR-2019-4324_supp.pdf]

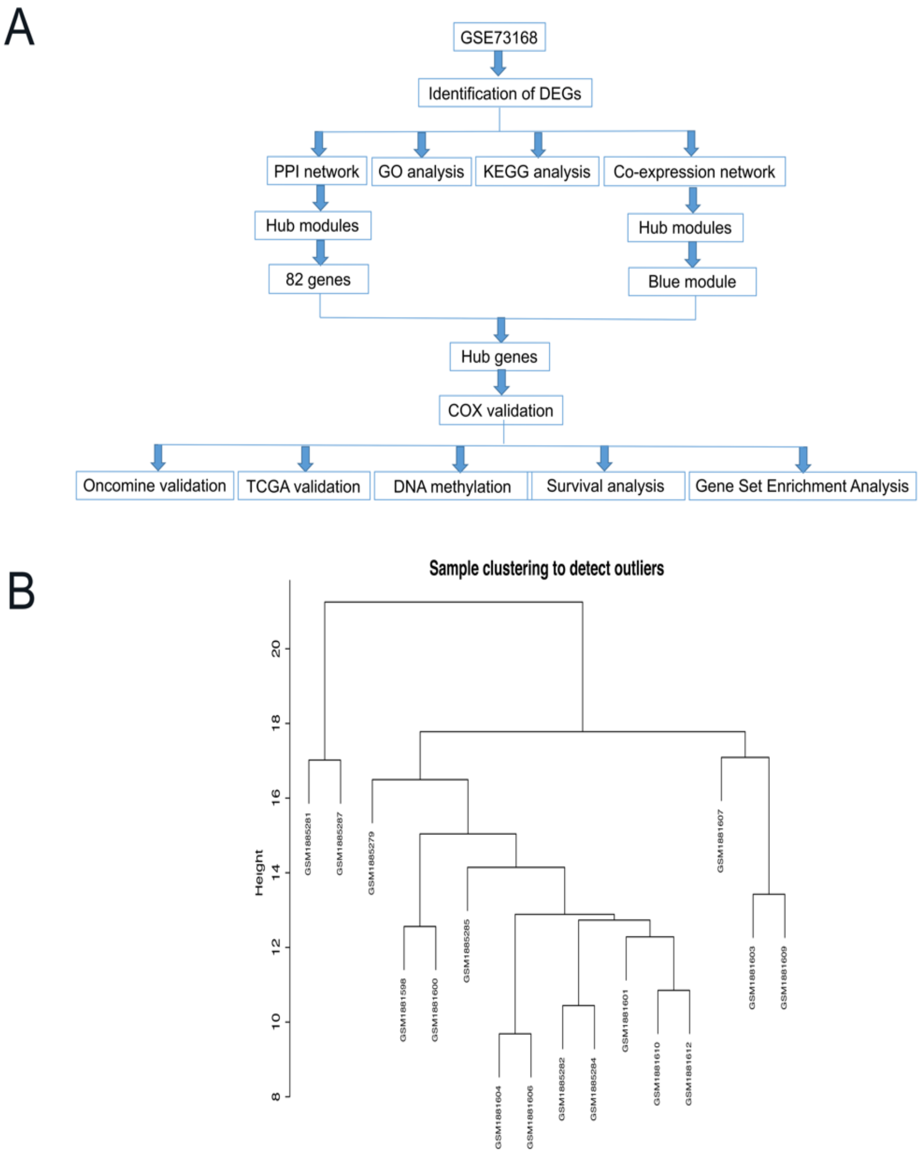

Fig.S1 Study design and data preprocessing. **A** Flow diagram of research. **B** Samples clustering of GSE73168 to detect outliers

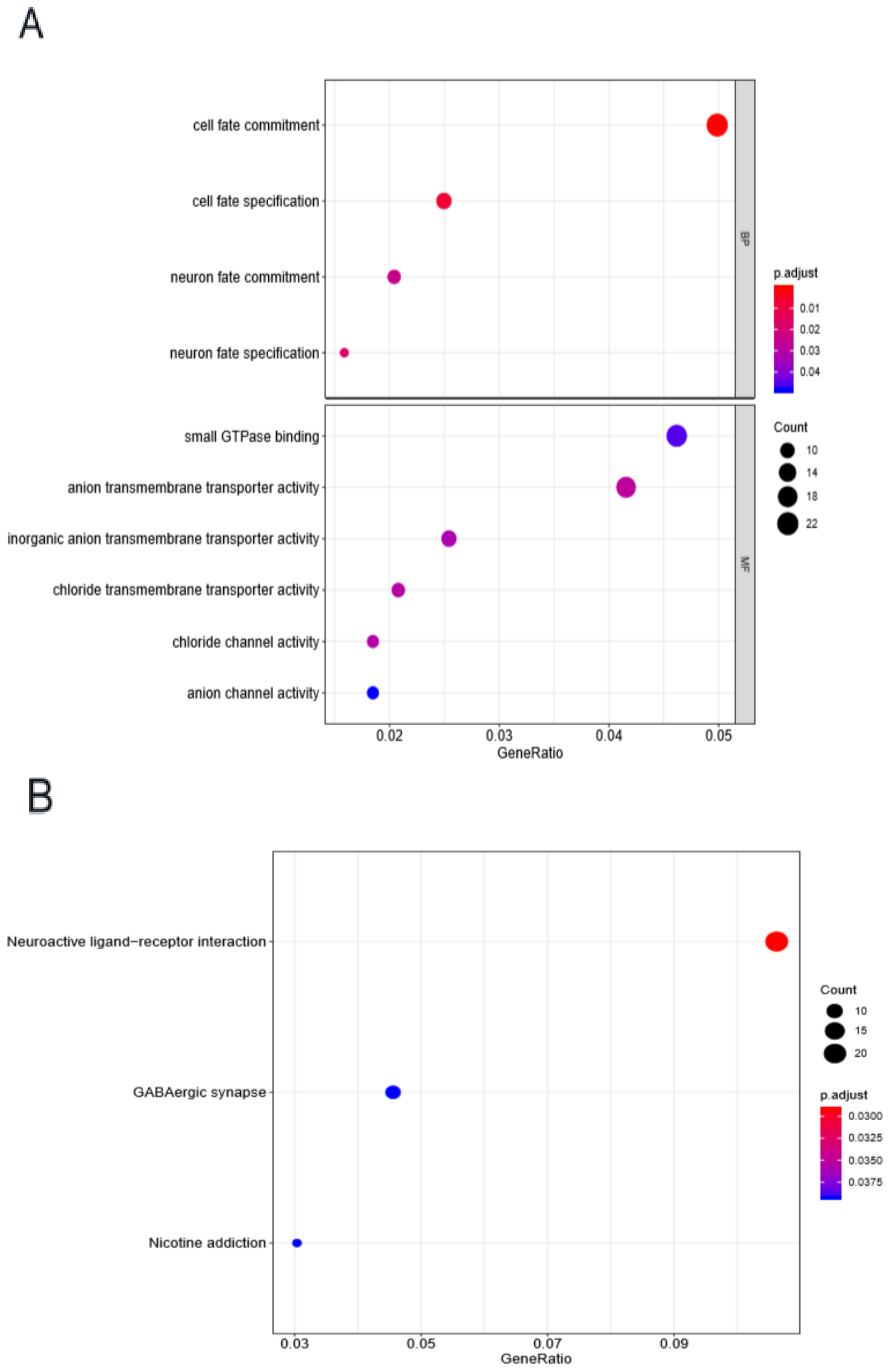

Fig. S2GO and KEGG enrichment analysis of DEGs.**A**GOenrichment analysis of DEGs. **B** KEGG enrichment analysis of DEGs.

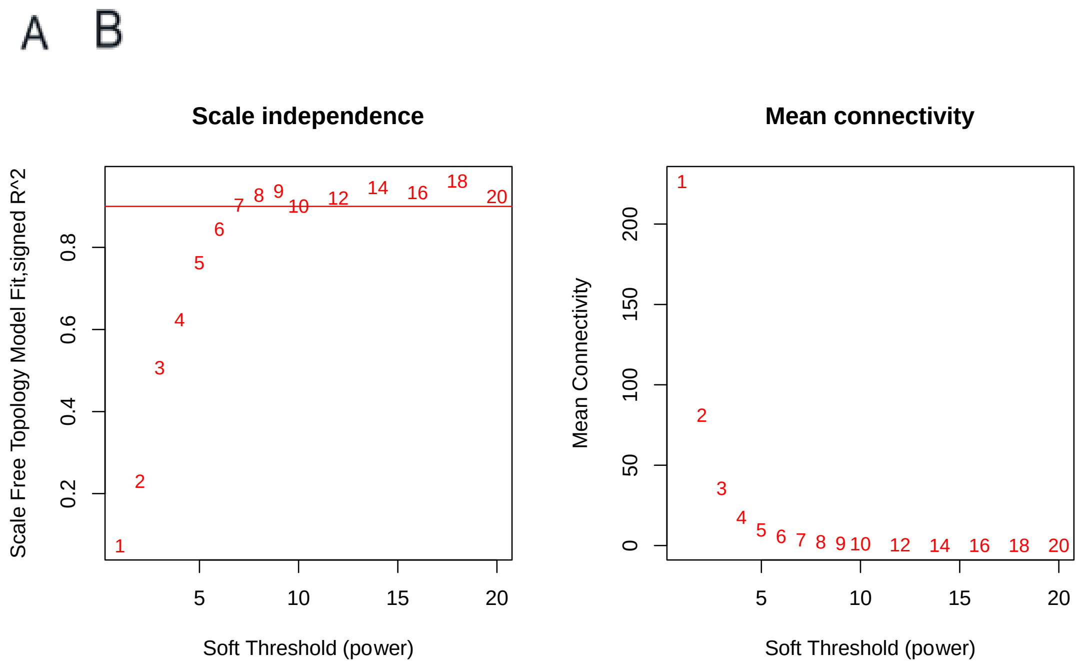

Fig. S3 Determination of soft-thresholding power in weighted gene co-expression network analysis and gene clustering dendrograms. **A** Analysis of the scale-free fit index for various soft-thresholding powers (β). **B**Analysis of the mean connectivity for various soft-thresholding powers.

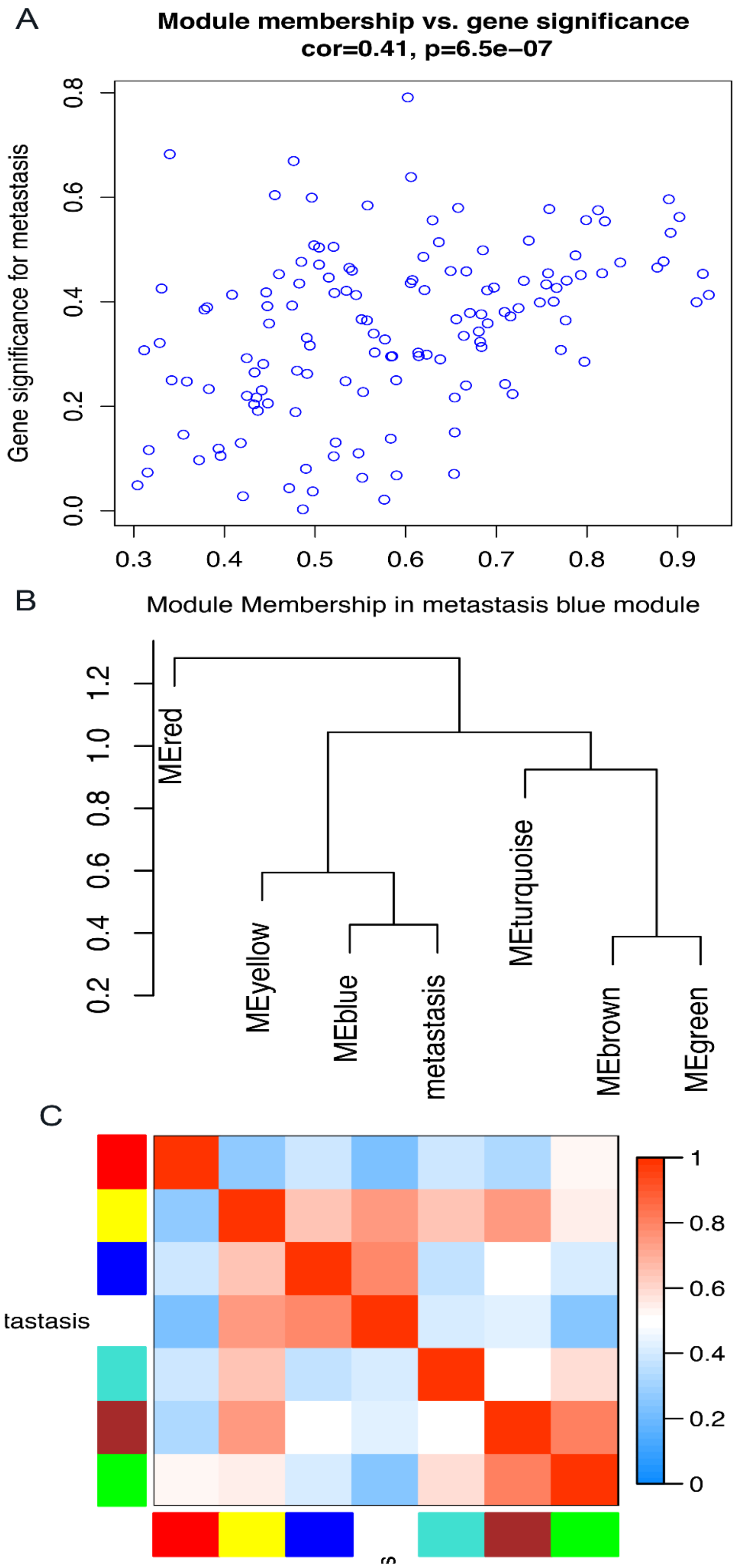
A
Module membership vs. gene significance
cor=0.41, p=6.5e−07
Gene significance for metastasis
Module Membership in metastasis blue module
0.0
0.2
0.4
0.6
0.8
0.3
0.4
0.5
0.6
0.7
0.8
0.9
B
MEred
MEyellow
MEblue
metastasis
MEturquoise
MEbrown
MEgreen
0.2
0.4
0.6
0.8
1.0
1.2
C
tastasis
0
0.2
0.4
0.6
0.8
1

Fig. S4 Select hub genes in hub modules. **A** A scatter plot of GS versus the MM in the blue module. Intramodular analysisof the genes found in the blue module. **B** Dendrogram of consensus module eigengenesobtained by WGCNA on the consensus correlation. **C** Heatmap plot ofthe adjacencies of modules. Red represents high adjacency (positivecorrelation) and blue represents low adjacency (negative correlation).

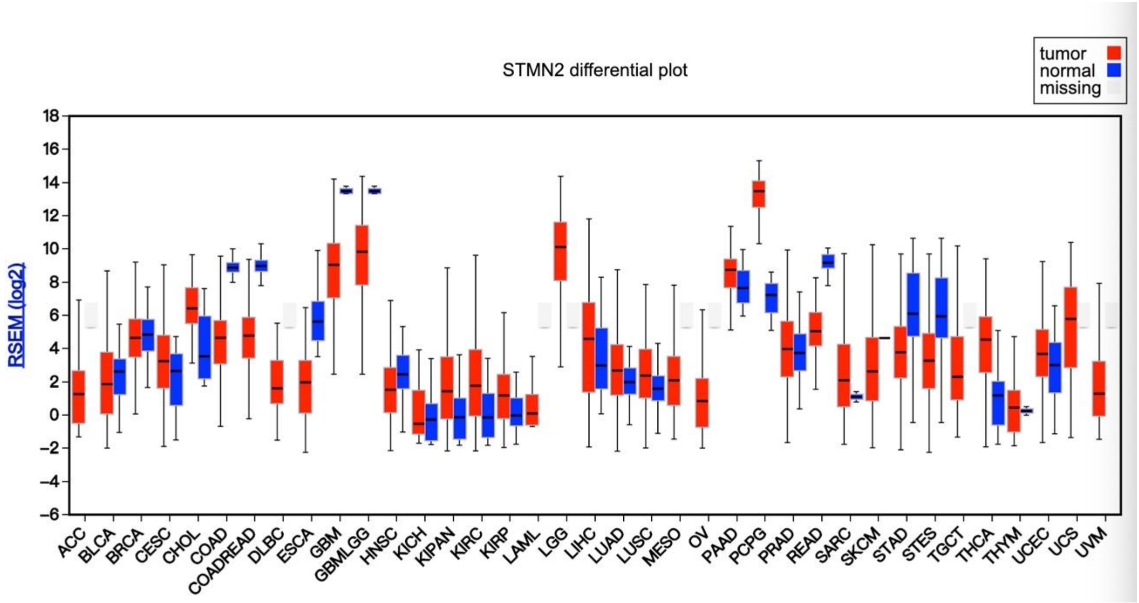

Fig. S5 Differential expression analysis of STMN2 in 37 types of human cancers on the Firebrowse website.

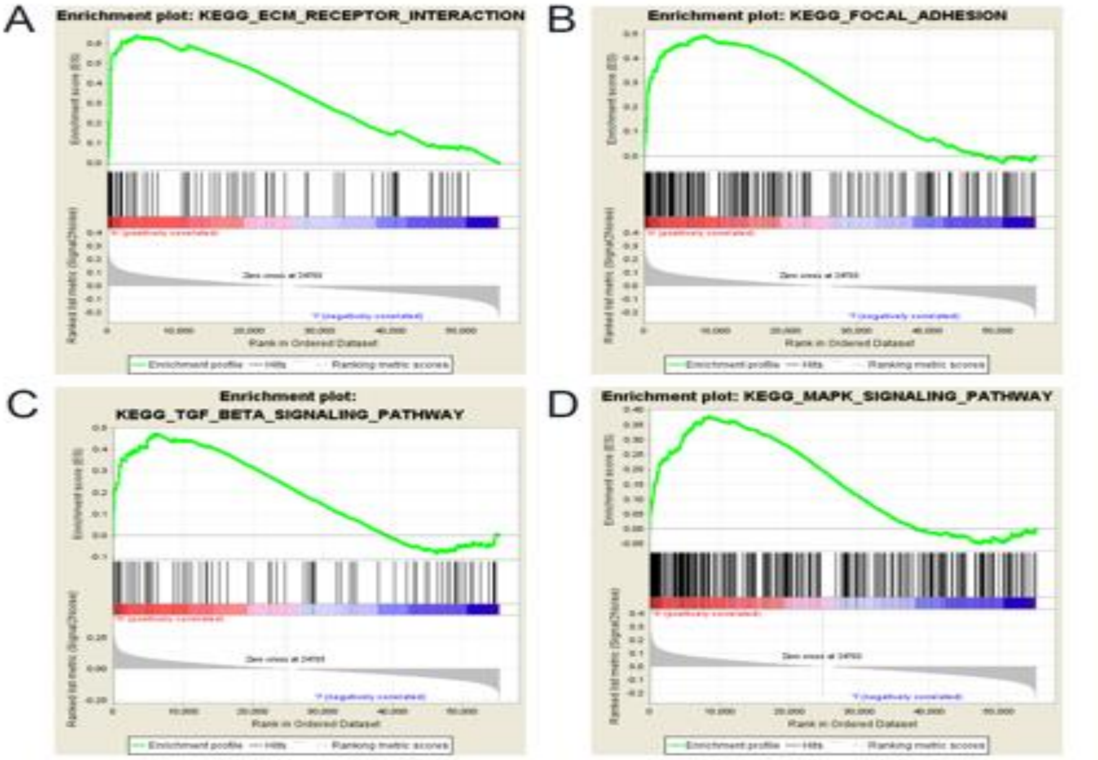

Fig. S6 Gene set enrichment analysis (GSEA) using GSE73168. Only listed the four most common functional gene sets enriched withhub genes highly expressed.**A**ECMreceptorinteraction**B**Focaladhesion**C**TGF-β signalingpathway**D**MAPKsignalingpathway

TableS1. Univariate Cox regression analysis of the remaining 16 genes and clinical features

	Univariate analysis		
Variables	HR	95% CI	P-value
Age	1.02	1.007-1.034	0.0036*
Grade	1.378	0.904-2.102	0.136
Stage	1.391	1.023-1.892	0.0351*
HOXA3	1.057	0.9280-1.203	0.4046
ISL1	0.893	0.537-1.485	0.6635
DSC3	1.081	0.951-1.229	0.2317
CCR3	0.734	0.273-1.972	0.5397
BEST1	0.843	0.386-1.844	0.6693
GABRB1	0.236	0.051-1.080	0.0627
TAS2R50	1.852	0.068-50.724	0.7152
FGF10	1.327	0.828-2.127	0.2392
HK2	1.059	0.876-1.278	0.5548
DCLK1	1.384	0.985-1.945	0.0608
LHX8	112.747	0.014-900634.862	0.3027
MMP24	1.053	0.916-1.210	0.4704
CD36	1.137	0.908-1.423	0.2628
ART4	2.711	0.139-52.923	0.5108
CLEC12A	0.751	0.533-1.058	0.1019
PTGER3	1.143	0.950-1.375	0.1567

HR,Hazard ratio

*P<0.05
